# Regulation of mouse hepatic genes in response to diet induced obesity, insulin resistance and fasting induced weight reduction

**DOI:** 10.1186/1743-7075-2-15

**Published:** 2005-06-28

**Authors:** R Michael Raab, John Bullen, Joanne Kelleher, Christos Mantzoros, Gregory Stephanopoulos

**Affiliations:** 1 Department of Chemical Engineering, Massachusetts Institute of Technology, Cambridge, MA, USA; 2 Beth-Israel Deaconess Medical Center, Harvard Medical School, Boston, MA, USA; 3 Department of Physiology, The George Washington School of Medicine and Health Sciences, Washington, DC, USA

## Abstract

**Background:**

Obesity is associated with insulin resistance that can often be improved by caloric restriction and weight reduction. Although many physiological changes accompanying insulin resistance and its treatment have been characterized, the genetic mechanisms linking obesity to insulin resistance are largely unknown. We used DNA microarrys and RT-PCR to investigate significant changes in hepatic gene transcription in insulin resistant, diet-induced obese (DIO)-C57/BL/6J mice and DIO-C57/BL/6J mice fasted for 48 hours, whose weights returned to baseline levels during these conditions.

**Results:**

Transcriptional profiling of hepatic mRNA revealed over 1900 genes that were significantly perturbed between control, DIO, and fasting/weight reduced DIO mice. From this set, our bioinformatics analysis identified 41 genes that rigorously discriminate these groups of mice. These genes are associated with molecular pathways involved in signal transduction, and protein metabolism and secretion. Of particular interest are genes that participate in pathways responsible for modulating insulin sensitivity. DIO altered expression of genes in directions that would be anticipated to antagonize insulin sensitivity, while fasting/ weight reduction partially or completely normalized their levels. Among these discriminatory genes, *Sh3kbp1 *and *RGS3*, may have special significance. *Sh3kbp1*, an endogenous inhibitor of PI-3-kinase, was upregulated by high-fat feeding, but normalized to control levels by fasting/weight reduction. Because insulin signaling occurs partially through PI-3-kinase, increased expression of *Sh3kbp1 *by DIO mice may contribute to hepatic insulin resistance via inhibition of PI-3-kinase. *RGS3*, a suppressor of G-protein coupled receptor generation of cAMP, was repressed by high-fat feeding, but partially normalized by fasting/weight reduction. Decreased expression of RGS3 may augment levels of cAMP and thereby contribute to increased, cAMP-induced, hepatic glucose output via phosphoenolpyruvate carboxykinase (PCK1), whose mRNA levels were also elevated.

**Conclusion:**

These findings demonstrate that hepatocytes respond to DIO and weight reduction by controlling gene transcription in a variety of important molecular pathways. Future studies that characterize the physiological significance of the identified genes in modulating energy homeostasis could provide a better understanding of the mechanisms linking DIO with insulin resistance.

## Background

Obesity is a growing concern in the industrialized world. It is estimated that over 61% of adult Americans are overweight or obese [1] and an alarming number of children and adolescents are following suit [2]. Of primary concern are the associated complications stemming from obesity's growing prevalence, among which type 2 diabetes is reaching epidemic proportions.

The aetiology of type 2 diabetes is complex because of its heterogeneous origins that result in the commonly observed hyperglycemia and hyperinsulinemia, which are characteristic of insulin resistance. While an enormous number of investigations have resulted in identifying some of the relevant molecular pathways, particularly in muscle and adipose tissue, more research is required to fully understand genetic susceptibility to type 2 diabetes and insulin resistance.

In the liver, hepatic glucose output (HGO) increases during insulin resistance and several key molecules contributing to this phenotype have been widely studied [3-6]. Despite these extensive efforts, the genes identified thus far do not alone account for all of the variability in HGO. Further insight may be obtained by conducting genome wide transcriptional studies during diet induced obesity (DIO) and its associated insulin resistant physiological state. This approach is a critical step towards further defining the molecular processes that regulate the phenotype and thereby augment the discovery of new potential therapeutic targets.

C57/BL/6J mice fed a high-fat diet become obese, hyperglycemic, and hyperinsulinemic, reflecting an insulin resistant metabolic state [7-11] that resembles the human condition. Although it has been demonstrated that short-term caloric restriction can improve insulin resistance [12], the regulatory pathways that control hepatic metabolism during DIO and associated insulin resistance, and the improvement of insulin resistance with caloric restriction, are the focus of intense research efforts. The molecular mechanisms underlying these pathways rely upon alterations in gene transcription [13], which can be monitored using DNA microarrays [14,15].

To investigate hepatic gene regulation in response to DIO and insulin resistance, whole genome microarrays containing 17,280 gene probes were used to examine transcription in two groups of C57/BL/6J mice : 1) the "control mice" received a normal diet for 10 weeks, 2) the "high-fat mice" received a high-fat diet for 10 weeks. In addition, to assess hepatic gene regulation in response to caloric restriction, which is a commonly recommended treatment for DIO and insulin resistance, a third group of mice was used, the "fasted/ weight reduced mice", which was fed the same high-fat diet for ten weeks followed immediately by 48 hours of fasting, returning their weights to baseline levels prior to tissue harvest. Fasting/ weight reduction data provides further differentiation among genes that not only respond to DIO and insulin resistance, but are also normalized by caloric restriction.

An extensive bioinformatics analysis led to the identification of 41 discriminatory genes participating in key molecular pathways in DIO, insulin resistance, and fasting/ weight reduction. The implicated pathways involve signal transduction and protein metabolism and secretion. In addition, the 41 genes identified can accurately classify the three groups of mice ("control", "high-fat", and "fasted/ weight reduce"), and importantly, they represent a set of candidate genes that may influence hepatic function during periods of insulin resistance and sensitivity.

## Methods

### Animals

Three to five week old C57/BL/6J mice were obtained from Jackson Laboratories (Bar Harbor, ME). All animals were allotted a seven day acclimation period with access to food and water ad libitum, and were maintained at 25°C with a 12-hour light/ dark cycle (lights on from 06:30–18:30) for the duration of the study. A normal chow (Purina Rodent Chow; Harlan Teklad #5008; 6.5% fat, 49% carbohydrate, 23% protein, 3.5 kcal/g) and high-fat diet (Harlan Teklad #TD88137, 42.16% fat, 42.81% carbohydrate, 15.02% protein, 4.53 kcal/g) were fed to respective mice, as outlined below.

This report explored alterations in hepatic gene mRNA levels in C57/BL/6J mice fed either a control or high-fat diet for 10 weeks, as well as alterations in mRNA levels of C57/BL/6J mice fasted for 48 hours following 10 weeks of high-fat feeding. Fasted animals were allowed access to water during the fasting period. All animals were sacrificed by CO_2 _asphyxiation, followed by immediate collection of liver tissue, which were stored at -80°C as previously described [16].

The control group consisted of C57/BL/6J mice fed normal chow diet for 10 weeks. The experimental group consisted of C57/BL/6J mice fed a high-fat diet for 10 weeks (n = 9/group). The ten week high-fat dietary treatment has been demonstrated to be long enough for C57/BL/6J mice to develop insulin resistance and a condition that resembles type 2 diabetes [7,8]. Two days before tissue harvest, the C57/BL/6J mice on the high-fat diet were divided into two groups, with one group remaining on the high-fat diet (n = 5; to be used in the first study) and one group fasting for the final 48 hours (n = 4; to be used in the second study). Mouse weights were recorded two days prior to, and on the day of tissue harvest. All animals were handled in accordance with the principles and guidelines established by the National Institutes of Health. The protocol was approved by the Institutional Review Board at Beth Israel Deaconess Medical Center, Boston, MA.

### Preparation of total RNA and cDNA for microarray hybridization

Total RNA was purified from liver tissue samples using STAT-60 (Tel-Test, Inc., Friendswood, TX) according to the manufacturer's instructions, and stored at -80°C. Labeled control cDNA was made from Total RNA control samples (Universal Mouse Reference RNA, catalog #740100, Stratagene) using Cy3 dCTP (Perkin-Elmer), and labeled liver cDNA was made from total RNA experimental samples using Cy5 dCTP (Perkin-Elmer) during reverse transcription, as described previously [17].

Microarrays were prepared using GAPS glass slides (Corning) and a Virtek arrayer (Bio-Rad). Arrays contained 17,280 features, printed from a synthesized oligonucleotide mouse library (Operon) as described previously [17].

### RT-PCR analysis of IL6st, PTP4a2, G6P, PCK1, and malic enzyme

A two-step RT-PCR protocol was performed to confirm the mRNA levels of several genes. In this procedure the cDNA synthesis was performed as detailed previously [17] except the Cy-labeled nucleotides were replaced with unlabeled nucleotides such that all dNTPs were at the same final concentration during the reaction. PCR was conducted in 94-well plates using the iQ SYBR Green Supermix Kit (Bio-Rad), according to the manufacturer's instructions on an iCycler RT-PCR machine (Bio-Rad). Briey, 1 *μ*L of the final, diluted cDNA template was mixed with 19 *μ*L of RNase free water, 25 *μ*L of Bio-Rad RT-PCR Supermix (Bio-Rad), 2 *μ*L of sense and antisense primers, and 1 *μ*L of 12.5 mM dNTPs. The final primer concentration was 0.25 *μ*M. The PCR cycle used a single three minute hot-start at 95°C, followed by 50 cycles of 30 seconds at 95°C, one minute at 60°C, and two minutes at 72°C during which time the reaction fluorescence was measured. Each mouse sample was measured in either triplicate or quadruplicate. The sense and antisense primer sequences were: for interleukin 6 signal transducer *IL6st *5'-GCGGCTCGAACTTCACTGC-3', and 5'-CACGATGTAGCTGGCATTCACG-3'; for protein tyrosine phosphatase 4a2 *PTP4a2 5*'-TTTCTGCTGCGGAACATTTCAAG-3', and 5'-GCGTGCGTGTGTGAGTGTG-3'; for regulator of g-protein signalling 3 *RGS3 *5'-GCACATCCCGCATTCCAGTTAC-3', and 5'-AGGGAACACCAGGACTTTAGGG-3'; for glucose-6-phosphatase *G6P *5'-GTGATTGCTGACCTGAGGAACG-3', and 5'-TGCCACCCAGAGGAGATTGATG-3'; for phosphoenolpyruvate carboxykinase *PCK1 *5'-CAGAGAGACACAGTGCCCATCC-3', and 5'-AAGTCCTCTTCCGACATCCAGC-3'; for malic enzyme 5'-GCCAGAGGATGTCGTCAAGG-3', and 5'-ATTACAGCCAAGGTCTCCCAAG-3', respectively. These primers each gave specific fragments of the correct length when viewed upon a 4% agarose gel (data not shown). As an internal control *β*-Actin mRNA levels were also measured. The sense and antisense sequences were 5'-AATAAGTGGTTACAGGAAGTC-3' and 5'-ATGAAGTATTAAGGCGGAAG-3', respectively.

Gene specific standards were developed by amplifying the entire mRNA coding sequence of each gene by PCR, gel purifying the resulting band, and then diluting it to concentrations from 10^4 ^*μ*g/*μ*L to 10 9 *μ*g/*μ*L. The R^2 ^value of the standard curve, relating the threshold cycle to the amount of standard template, was always greater than 0.97. The mRNA levels of *β*-actin measured were not significantly (*p *> 0.05) different between the dietary treatments for any of the groups.

### Array validation

Microarray protocols have been extensively validated in our laboratory as described previously [17]. For validation, we prepared arrays containing an approximately 13,000 gene sub-set of our oligonucleotide mouse library, printed in triplicate. Total RNA from skeletal muscle and brain tissue were used for validation comparisons, and each sample was analyzed in duplicate and prepared and processed as described above. Matlab was used to calculate basic statistics.

The arrays' ability to detect differential transcription between muscle and brain RNA was evaluated by two different methods. In the first, we examined the number of genes that were up- or down-regulated by a factor greater than two (i.e., whose mean ratio was either greater than two, or less than 0.5) in the muscle versus muscle and the muscle versus brain RNA comparisons. This criterion has been used as a basis for assessing differential transcription in a number of studies [18-20]. In the second method, we defined a threshold for differential expression by using the 95% confidence interval determined from the muscle versus muscle control arrays. Table 1 summarizes the results, where the p-values reported were from two-tailed student t-tests.

**Table 1:** Differential gene transcription validation data. This table summarizes the results of the array validation with respect to the study of differential expression.

Array Condition	# of Probes Detected	# of Genes >2-Fold Different	Differentially Genes at the Expressed 95% Confidence Level
Muscle vs. Muscle	7574	438	429
Muscle vs. Muscle	6417	314	302
Average	6996	376	366

Muscle vs. Brain	7143	1201	1161
Muscle vs. Brain	8318	981	931
Average	7731	1091	1046

P-value	0.47	0.03	0.03

Although there are only about 370 genes exceeding the threshold in the muscle versus muscle arrays, more than 1000 genes were differentially expressed in the muscle versus brain arrays. This result supports the assertion that the assaying method and selection criterion are significantly more likely to identify differentially expressed genes.

**Figure 1:**
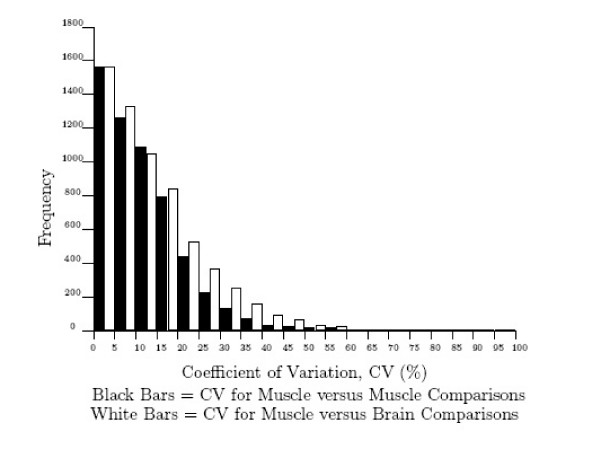
Distribution of the coefficient of variation for DNA microarrays. The coefficient of variation was calculated for every gene in the experiment, and plotted for the muscle versus muscle and muscle versus brain.

The coefficient of variation, CV, was calculated for each replicated gene expression and the distribution across all genes is plotted in Figure 1. For the muscle versus muscle control arrays, the median CV across all probes was 10.2%. For the muscle versus brain arrays the median coefficient of variation across all probes was 9.8%. This indicates that for a gene transcription ratio of 1, we might expect the true value to lie between 0.9 and 1.1; similarly for a gene transcription ratio of 3, we might expect the true value to lie between 2.7 and 3.3. Although the median CV across all probes for the muscle versus muscle control arrays was 10.2%, the median CV for the 314 genes common to both muscle versus muscle arrays that had a fold difference greater than two, was 24.7%. Because of their increased CV and high fold change, none of these genes were included in our subsequent analysis.

In duplicate arrays, 76% of the genes observed on one muscle versus muscle array were also observed on the duplicate; likewise 77% of the genes found on one muscle versus brain array were conserved on the duplicate. These data demonstrate the inter-array reproducibility by showing the majority of genes are reproducibly found in multiple replicate arrays.

RT-PCR was also used to verify the array results for *IL6st*, *PTP4a2*, and *RGS3*. The variation in the ratios of the mRNA levels was less than 30% for each of these genes whether measured using the arrays or RT-PCR as shown in Table 2.

**Table 2:** Comparison of array results and RT-PCR results for selected genes. Gene expression percentages are reported relative to the control values. F/ WR: Fasting Weight Reduction.

Genes	Assay	High Fat vs. Control	F/ WR vs. Control
*IL6st*	Array	154 ± 21%*	144 ± 21%*^†^
	RT-PCR	167 ± 19%*	185 ± 15%*^†^

*PTP4a2*	Array	71 ± 4%*	89 ± 3%*
	RT-PCR	75 ± 16%	94 ± 18%

*RGS3*	Array	35 ± 5%*	54 ± 8%*
	RT-PCR	38 ± 9%*	59 ± 8%*

*G6P*	RT-PCR	476 ± 72%	769 ± 216%*

*PCK1*	RT-PCR	132 ± 28%	217 ± 80%*

*Malic Enzyme*	RT-PCR	9.1 ± 1.5%*	0.1 ± 0.1%*

*Indicates that the measurements were significantly different from control values at P < 0.01.

^†^Indicates that the measurements made on the micro array were significantly different from the RT-PCR measurement at P < 0.05.

### Computational methods

A combination of statistical and data mining methods were used to extract information from the microarray data. Statistical methods rigorously quantify the reliability of differences in the microarray data [21] and can objectively evaluate changes in gene transcription ratios and derivative quantities. Data mining is particularly useful for uncovering patterns and structure in microarray data that might have otherwise been difficult to detect through manual inspection and intuition alone [22,23]. Applying statistics and data mining methods to microarray data in unison enables rapid and reliable analysis without *a priori *assumptions that may bias expectations about the data set.

A t-test [24] was used to evaluate whether a gene exhibited statistically significant expression differences in pairwise comparisons between the control, high-fat, and fasting/ weight reduced groups. The t-test results showed that 1981 genes had at least one statistically significant (*p *< 0.05) change between the treatments. Wilks-*λ *based ranking [25] was used to identify discriminatory genes that differentiated the three groups. This technique is particularly appropriate for *multi-class *comparisons, ranking genes on the basis of their within group, and between group variances. Thus, a gene exhibiting a small variation within each of the three groups, but a large variation between groups would rank highly; conversely, a gene that had a high level of variation within each group and a low level of variation among the groups would be ranked low. The Wilks-*λ *score can be transformed into an *F *statistic, which can be compared with the *F *distribution to assess the statistical significance of the observation [25]. In this analysis a Wilks-*λ *threshold value of 0.47 was used, which is equivalent to a p value of 0.05. From the 1981 genes selected by the *p *< 0.05 cutoff, we retained the 1169 genes that had a Wilks-*λ *value below 0.47.

**Figure 2:**
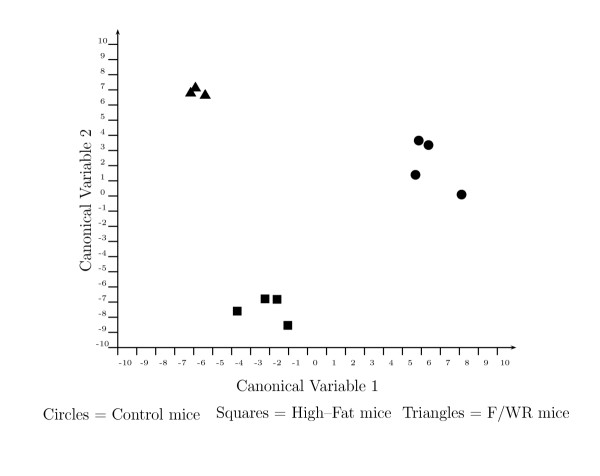
Fisher discriminant analysis plot of mouse liver samples. Samples were scored according to the canonical variables determined by Fisher Discriminant Analysis (FDA). Each canonical variable is defined as a weighted sum of 100 specific genes, including each of the 41 genes contained in Table 3 (See Additional file: 1). To score a sample, the gene expression value is multiplied by an FDA coefficient, called a loading, and the products from the 100 genes used in the analysis are summed to give the canonical variable score for the sample. F/ WR: Fasting/ Weight Reduced.

Fischer Discriminant Analysis [26] (FDA) was used to identify not just individual genes, but combinations of genes whose expression levels are capable of correctly classifying the control mice, high-fat mice, and fasting/ weight reduced mice. FDA is based on *linear combinations of gene expressions *and considers the discriminatory power of gene groups as opposed to individual genes. Samples are scored based on the weighted contribution of each gene's expression level to a newly defined metric called a "canonical variable" (CV). Because each gene's contribution to a sample's score is weighted by a coefficient called a "loading," genes with very small loadings do not significantly contribute to the sample's score and classification, and can therefore be eliminated from further consideration. This technique can be used as a tool to visualize the gene transcription results in a lower dimensional space defined by the canonical variables. As shown in Figure 2, using expression data of the selected gene combinations allows accurate classification of the dietary treatments suggesting that the genes in Table 3 (See Additional file: 1) are discriminatory of the conditions examined when sample classification is used as a criterion. On the basis of the successful classification afforded by the FDA projection, discriminatory genes were selected using the magnitude of the loading coefficients. Principle Component Analysis [27] was used as an unsupervised classification procedure to complement FDA. The results of the PCA analysis largely mirrored the FDA results (data not shown).

Methods used here, along with the data set, are available for public use at our laboratory's web-site [28]. The entire data set is also available through the National Center for Biotechnology Information's Gene Expression Omnibus database [29].

## Results

### The effect of 10 weeks of high-fat feeding and 48 hours of caloric restriction on body weight in C57/BL/6J mice

C57/BL/6J mice significantly increased their body weight by 32% after 10 weeks of high-fat feeding (*p *< 0.001; Table 4). After 48 hours of fasting, their weights returned to baseline levels and were not significantly different from the control mice, but were significantly less than mice maintained on the high-fat diet (*p *< 0.001; Table 4).

**Table 4:** Experimental treatments and mouse weights.

Diet	Feeding Regimen	Weight 48 hours Prior to Harvest (Average ± St. Dev., n)	Weight at Harvest (Average ± St. Dev., n)
Normal Chow	Ad libitum	35.6 ± 1.8, 9	35.6 ± 1.5, 9
High-Fat	Ad libitum	47.1 ± 5.8*, 9	51.7 ± 4.4*^†^,5
High-Fat	Restricted		37.3 ± 2.6,4

*Indicates that the weight was statistically different from the control at P < 0.001.

^† ^Indicates that the weight of the high-fat and fasted mice was different at P < 0.001.

### Microarray analysis of hepatic genes after 10 weeks of high-fat feeding and 48 hours of fasting/ weight reduction in C57/BL/6J mice

Employing statistical and data mining methods we searched the transcription data set for hepatic genes that direct the biological response during DIO, associated insulin resistance, and fasting/ weight reduction. We used the t-test to determine the statistical significance of every pairwise gene difference between the treatments. The t-test showed that 1981 genes had at least one statistically significant (*p *< 0.05) change between the treatments. Within this gene set, 113 genes were significantly changed between the high-fat fed mice and the control mice, 169 genes were significantly changed between the fasting/ weight reduced mice and the control mice, and 260 genes were significantly changed between the high-fat fed and fasting/ weight reduced mice, all at *p *< 0.01. From the 1981 genes selected by the *p *< 0.05 cutoff, we retained the 1169 genes that had a Wilks-*λ *value below our cutoff criterion of 0.47, which is equivalent to a p-value of less than 0.05 [25]. From these genes we selected those with the greatest Fisher Discriminant Analysis (FDA) and Principle Component Analysis (PCA) loading coefficients [27], resulting in the 41 genes reported in Table 3 (See Additional file: 1).

The 41 discriminating genes contributed to the classification observed in Figure 2. In Figure 2, each sample is given a canonical variable (CV) score, based on the weighted sum of its gene expression values. The genes with the largest contributions to CV1 and CV2 are given in Table 3 (See Additional file: 1), suggesting these genes underlie the biological differences between the samples. Figure 2 shows that 10 weeks of high-fat feeding altered the transcriptional levels of genes composing CV1 and CV2 so as to separate the control and high-fat mice in the CV1 and CV2 space. However, while 48 hours of fasting/ weight reduction normalized many of the genes contributing to CV2, resulting in a return to control levels for that variable, the genes contributing to CV1 remained perturbed, resulting in the observed separation between the fasted/ weight reduced mice and control mice. This suggests that while some genes, and their associated pathways that differentiate DIO and insulin resistance from normal physiology, return to control levels as weight is reduced, other genes remain perturbed, reflecting further physiological adaptations that occur during these treatments. To show individual gene responses to the dietary treatments, the 41 genes were clustered according to changes in the p-values from pairwise comparisons between the control mice, the high-fat fed mice, and the fasting/ weight reduced mice. This classification arranges the genes according to their transcript levels during the physiological states examined. For example, Group A in Table 3 (See Additional file: 1) comprises genes that were significantly elevated or repressed (*p *< 0.05) by high-fat feeding, but then normalized to (insignificant, *p *> 0.05) control levels by fasting and weight reduction. Similarly, group B genes were significantly elevated or repressed (*p *< 0.05) by high-fat feeding and partially normalized to control levels by fasting/ weight reduction: the expression differences are still significant (*p *< 0.05) when comparing both the high-fat and control mice with the fasted/ weight reduced mice. The genes of each group along with their normalized expression levels are given in Table 3 (See Additional file: 1).

Among the 41 discriminatory genes identified in this study, interleukin 6 signal transducer (*IL6st*), protein tyrosine phosphatase 4a2 (*PTP4a2*), SH3-domain kinase binding protein 1 (*Shk3bp1*), and regulator of g-protein signaling 3 (*RGS3*) are of special interest because, based on known biology, they may contribute to the physiological changes that accompany DIO, insulin resistance, and increased insulin sensitivity due to fasting/ weight reduction. Both *IL6st *and *Sh3kbp1 *are significantly upregulated after 10 weeks of high-fat feeding (*p *< 0.001), but only *Sh3kbp1 *is normalized to baseline levels after 48 hours of fasting and weight reduction (Table 3: See Additional file: 1). Both *PTP4a2 *and *RGS3 *are significantly downregulated after 10 weeks of high-fat feeding (*p *< 0.01), and both are partially normalized after 48 hours of fasting/ weight reduction (*p *< 0.01 for fasted/ weight reduced versus high-fat and fasted/ weight reduced versus control; Table 3: See Additional file: 1).

### RT-PCR analysis of IL6st, PTP4a2, RGS3, G6P, PCK1, and malic enzyme

We compared the transcript levels measured by RT-PCR with the ratios measured using DNA microarrays by dividing RT-PCR expression values observed in high-fat fed mice and fasted/ weight reduced mice by the expression values measured in the control mice. Liver mRNA levels for each mouse in the study were determined by RT-PCR for *IL6st*, *PTP4a2*, and *RGS3*. The values measured by RT-PCR were not significantly different from the results observed by hepatic microarray analysis (*p *> 0.05; Table 2) for all genes except *IL6st *between the fasting/ weight reduced mice and control mice. Notably, in this single case, both microarray analysis and RT-PCR show significant increases (*p *< 0.001) in the levels of *IL6st *mRNA, demonstrating similar qualitative changes between the measurement methods. The close agreement between the micoarray results and RT-PCR results thus validates the specificity and accuracy of our microarray measurements. The difference in the ratios between the values determined by RT-PCR and those determined by microarray analysis was less than 30% for each of these genes (Table 2).

Although several commonly studied genes, such as glucose-6-phosphatase (*G6P*), phosphoenolpyruvate carboxykinase (*PCK1*), and malic enzyme, did not make it into our bioinformatics analysis, we evaluated their expression by RT-PCR because of their considerable effects on hepatic glucose output. *G6P *and *PCK1 *were upregulated following 10 weeks of high-fat feeding, but only the change observed in *G6P *achieved statistical significance (*p *= 0.09 for *PCK1 *and *p *< 0.01 for *G6P *in the high-fat versus control comparison; Table 2). Fasting/ weight reduction resulted in even larger increases in mRNA levels for both *G6P *and *PCK1 *(*p *< 0.01 versus controls; Table 2). In contrast, malic enzyme exhibited significant underexpression following 10 weeks of high-fat feeding, with further down-regulation following fasting/ weight reduction (Table 2).

## Discussion

Diet induced obesity (DIO) in C57/BL/6J mice is a commonly used animal model for the development of insulin resistance in humans [7-11], which results in simultaneous hyperglycemia and hyperinsulinemia. Although short-term caloric restriction and weight loss can improve insulin resistance [12,30,31], the regulatory mechanisms in the liver that lead to insulin resistance in response to DIO, as well as the improvement of insulin sensitivity in response to short-term caloric restriction and weight reduction, remain largely unknown. To identify genes involved in hepatic physiology during DIO and short-term caloric restriction, we used DNA microarrays to measure genome-wide transcript abundance.

The 41 most discriminating genes determined by our bioinformatics analysis lie essentially within two large groups (Table 3: See Additional file: 1): 1) Genes that are significantly induced or repressed by 10 weeks of high-fat feeding and completely (Group A) or partially (Group B) normalized by 48 hours of fasting/ weight reduction, 2) Genes that are significantly induced or repressed by 10 weeks of high-fat feeding, but are not normalized by 48 hours of fasting/ weight reduction (Group D). Both of these groups contain genes involved in signal transduction pathways, as well as protein metabolism and secretion, highlighting the importance of these molecular pathways in the hepatic response to DIO and fasting/ weight reduction.

Because genes in Group A and B (Table 3: See Additional file: 1) were perturbed by DIO, their expression levels correlate with observed physiological differences that develop during this condition. These differences include elevated concentrations of serum triglycerides, leptin, and tumor necrosis factor-*α*, as well as changes in the levels of other factors that have been previously demonstrated to play a physiological role during DIO in C57/BL/6J mice [7-9,11,32]. Notably, Group A and B genes are either completely (Group A, Table 3: See Additional file: 1) or partially (Group B, Table 3: See Additional file: 1) normalized following 48 hours of fasting/ weight reduction, when insulin sensitivity has increased, suggesting they may be important to the development of hepatic insulin resistance during DIO. Several relevant signal transduction pathways are influenced by the genes within Group A and B (Table 3: See Additional file: 1), particularly *Sh3kbp1*, *PTP4a2*, and *RGS3*. While *Sh3kbp1 *and *PTP4a2 *may be directly involved with insulin signaling, by respectively binding PI-3-kinase and dephosphorylating protein tyrosine residues, *RGS3 *interacts directly with G-proteins and some evidence suggests RGS family members may also indirectly affect proteins in the MAPK signal transduction pathways [33] as well as certain tyrosine phosphatases [34].

**Figure 3:**
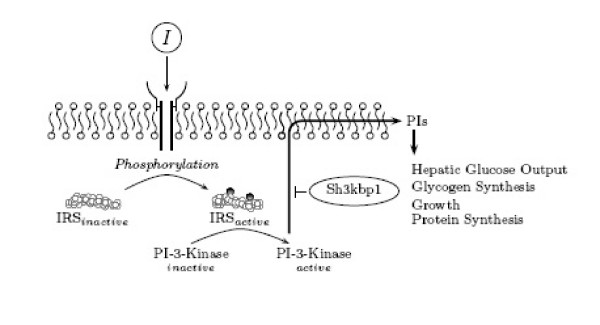
Inhibition of PI-3-Kinase signaling by Sh3kbp1. In this figure, insulin, *I*, binds to its receptor, activating the receptor's tyrosine kinase activity. Insulin receptor substrates, IRS, are activated by phosphorylation. IRS phosphorylates PI-3-kinase, which migrates to the cell membrane where it generates phosphatidylinositol, PI, second messengers, which alters physiological processes. Shown here, Sh3kbp1 is capable of binding the regulatory subunit of PI-3-kinase, inhibiting its ability to generate PI second messengers, and thereby attenuating insulin signaling.

*Sh3kbp1 *(SH3-domain kinase binding protein, also called Ruk) belongs to the CD2AP/CMS family of adapter-type proteins, which mediate a number of different cellular mechanisms including signal transduction [35]. Insulin signaling occurs via phosphorylation of insulin receptor substrates (IRSs) that interact with signal transduction molecules including PI-3-kinase, Grb2, nck, and SHP2 [36]. Sh3kbp1 has been shown to directly inhibit PI-3-kinase signaling by binding the p85*α *regulatory subunit *in vivo *and *in vitro*, and interacts with Grb2 *in vitro *[37]. Therefore, increased levels of *Sh3kbp1 *mRNA in the high-fat fed mice relative to both the control and fasted/ weight reduced mice, suggests that Sh3kbp1 may mediate DIO associated insulin resistance in hepatocytes via a mechanism described in Figure 3.

*PTP4a2 *(Protein tyrosine phosphatase 4a2) dephosphorylates tyrosine residues in proteins. When insulin binds its receptor it activates the receptor's tyrosine kinase activity [38], leading to autophosphorylation and subsequent tyrosine phosphorylation of molecules containing Src homology 2 (SH2) or phosphotyrosine binding (PTB) domains. Therefore PTPs can in fluence insulin signaling by dephosphorylating protein tyrosine residues. Although it would be anticipated that PTPs would attenuate insulin signaling, they have been implicated in both positive and negative regulation of this pathway [39]. A definitive role for many PTPs in glucose homeostasis and insulin signaling has not been established, however, *PTP1B *knock-out mice have enhanced insulin sensitivity and are resistant to DIO [40]. Therefore if PTP4a2 also negatively regulates insulin signaling, its significant downregulation (*p *< 0.01) following 10 weeks of high-fat feeding may be a physiological adaptation that protects hepatocytes against insulin resistance, which is normalized by fasting/ weight reduction.

*RGS3 *(Regulator of G-protein coupled receptor (GPCR) signaling 3) has been primarily studied in neurons [41-43] and cells in culture [44,45]. RGS proteins bind G*α *subunits and generally increase the GTPase activity [46]. We found that hepatic *RGS3 *mRNA levels are significantly decreased (*p *< 0.01) after 10 weeks of high-fat feeding, but partially normalized by fasting/ weight reduction. These findings are particularly relevant because hepatocytes express a truncated form of RGS3 that has been shown to directly inhibit G_*s*_*α *stimulated cAMP production and G_*q*_*α *stimulated IP production [47], in addition to interacting with, G_*i*_*α *[48]. Glucagon signals via a GPCR that stimulates adenyl cyclase and increases cAMP levels [49]. Because the truncated form of RGS3 inhibits cAMP production, lowering RGS3 concentration may augment basal cAMP levels and thereby promote hepatic glucose output resulting from cAMP induced phosphoenolpyruvate carboxykinase (PCK1) expression and cAMP repressed glucokinase transcription. Although glucokinase expression levels were not measured, *PCK1 *mRNA levels were increased by both 10 weeks of high-fat feeding and fasting/ weight reduction (Table 2).

While genes in Group D (Table 3: See Additional file: 1) were also significantly induced or repressed following 10 weeks of high-fat feeding, unlike genes in Group A and B, they do not respond to 48 hours of fasting/ weight reduction. Therefore hepatic regulation of Group D genes may not be as directly linked to changes resulting from DIO and insulin resistance and sensitivity. Despite this, it is interesting that a number of Group D genes are also implicated in several signal transduction pathways that may be activated by DIO. These genes include *BMP2*, *Fosb*, *Gabrr1*, *IL6st*, and *4833414G15Rik*.

*BMP2 *(Bone morphogenetic protein 2), is a highly conserved member of the transforming growth factor-*β *(TGF-*β*) gene family. BMP2 is related to BMP9, which was the first reported hepatic factor shown to decrease blood glucose levels by increasing insulin release and decreasing food intake [50]. While these mechanisms may be a compensating response to DIO, they oppose the physiological adaptations that accompany 48 hours of fasting/ weight reduction, and therefore additional studies are required to determine the effects of BMP2 upregulation in mice following these dietary treatments.

*FosB *is a member of the AP-1 family of transcription factors [51]. These molecules are considered immediate early genes, because they initiate responses to environmental stimuli [52]. The Fos family of transcription factors form either homodimers with one another, or heterodimers with the Jun family of transcription factors, which then bind DNA to alter gene transcription [53]. Because insulin affects the expression of members of the AP-1 family of transcription factors [54], it is not surprising that during DIO and fasting/ weight reduction, conditions that perturb insulin signaling, significantly increase transcription of *FosB*.

*IL6st *(Interleukin 6 signal transducing subunit, also called gp130) is a key component in cytokine signal transduction that occurs during inflammation through the JAK (Janus kinase)/ STAT (signal transducers and activators of transcription) pathway. IL6st forms homo- and heterodimers with other signal transducing subunits in response to binding by an assortment of ligands including IL-6, IL-11, LIF, CT-1, CNTF, and OSM [55]. Among these, IL-6 knockout mice develop mature-onset obesity [56], and treatment of hepatocytes with IL-6 reduces the expression of PCK1 [6], thus implicating IL-6 in the regulation of hepatic glucose output. There are at least four different Jaks (Jak1, Jak2, Jak3, and Tyk2) and seven different STAT factors (STAT1, 2, 3, 4, 5a, 5b, and 6) that can interact with IL6st. Of particular relevance to DIO and insulin resistance is STAT3. The liver-specific STAT3 knockout mouse is insulin resistant and develops glucose intolerance when fed a high-fat diet, due in part to increased expression of PCK1 and G6P [57]. Adenoviral mediated reconstitution of STAT3 signaling ameliorated glucose intolerance in both L-ST3KO and *Lepr*-/- mice [57] by lowering PCK1 and G6P levels, demonstrating the importance of STAT3 signalling to hepatic glucose output. Because *IL6st *is significantly upregulated (*p *< 0.001) by 10 weeks of high-fat feeding and 48 hours of fasting/ weight reduction, when *PCK1 *and *G6P *were also induced relative to control levels (Table 2), it may be that IL6st performs a sensitizing function that contributes to feedback control of hepatic glucose output via IL6 and STAT3 signaling. In addition to the cellular signaling pathways that contained differentially expressed genes identified in this study, a number of genes involved in protein metabolism and secretion were also identified. Although a direct link between protein metabolism/ secretion and DIO/ insulin resistance is not as well established, in other insulin sensitive tissues the release of hormones and trafficking of receptors clearly plays a role in regulating tissue specific responses to insulin and glucose. Group A and B genes involved in protein metabolism and secretion pathways include *Kcnk8*, *Pmm1*, *Serpina5*, and *Eif4a2*. Group D genes that were identified include *Copz2*, *Rab3c*, and *4933432M07Rik*.

*Serpina5*, encodes a serine protease inhibitor. Serine protease inhibitors represent a family of glycoproteins that are known to inactivate serine proteases by forming stoichiometric enzyme-inhibitor complexes. Among the proteases known to be inhibited by Serpins are trypsin, chymotrypsin, the sperm protease acrosin, and a variety of proteases involved in hemostasis [58]. *Copz2 *encodes a vesicle coating protein that helps to mediate vesicle trafficking, while *Rab3c *is a member of the Ras oncogene family that encodes a monomeric GTP-binding protein that is implicated in regulated exocytosis and vesicle transport, and has been suggested to play a role in GLUT4 translocation in rat cardiac muscle cells [59]. Hence, Copz2 and Rab3c may synergistically influence protein trafficking in response to 10 weeks of high-fat feeding and 48 hours of fasting/ weight reduction.

## Conclusion

Using DNA microarrays we have investigated the effects of DIO and fasting/ weight reduction on liver gene transcription. We have analyzed this data set using four computational methods that represent a rigorous approach to analysis requiring no *a priori *assumptions about the data. This has enabled us to infer the importance of any given gene change among a multitude of gene differences resulting from DIO and fasting/ weight reduction. Our results lead us to focus on 41, out of an initial 1981 genes.

Although many of the genes resulting from our analysis have not yet been studied extensively in the context of energy homeostasis, several are related to important molecular pathways that have been previously identified in the literature. Those pathways include different signal transduction cascades, as well as pathways involved in protein metabolism and secretion. Given the diverse functions of the liver, identifying genes involved in signaling and protein metabolism pathways in response to DIO and fasting/ weight reduction is not surprising. Among the genes involved in signaling are *Sh3kbp1*, *Rgs3*, *PTP4a2*, *BMP2*, *IL6st*, *Fosb*, *Gabrr1*, and possibly *Rab3c*. Genes implicated in protein metabolism and secretion pathways include *Crym*, *Serpina5*, *Eif4a2*, *Ctrl*, *Snrpg*, *Kcnk8*, *Copz2*, and *Rab3c*.

While the link between many of these genes and DIO will require further investigations, their identification here is an important contribution to understanding how the hepatic response to DIO and fasting/ weight reduction is mediated through a variety of molecular pathways. These genes all share a consistent set of attributes that made them stand out in the data set. They demonstrate significant differences between the dietary treatments, are individually discriminatory of each treatment, and are members of a set that classifies each sample using both supervised and unsupervised algorithms. Genes that satisfy all of these criteria represent good candidates for influencing the liver's response to DIO and fasting/ weight reduction, and therefore warrant more detailed investigations.

## Competing interests

The author(s) declare that they have no competing interests.

## Authors' contributions

RMR helped with the tissue harvest, conducted the RNA purification, prepared the DNA microarrays, conducted the sample labeling and hybridization, conducted the RT-PCR, conducted the data analysis, and wrote the manuscript. JB handled the animals used in the study, helped conduct the tissue and RNA harvests, and helped write the manuscript. JK consulted on the project and reviewed the manuscript. CM helped design the experiment and set-up the animal handling procedures. GS organized the project, helped design the experiments, and helped write the manuscript.

## Note

Table 3: (See Additional file: 1) Percent control expression of genes found common to all analysis methods. Included are genes identified using t-test, Wilks ranking, fisher discriminate analysis, and principle component analysis. These genes are organized by their pairwise t-test results, and the relation between their expression levels. F/ WR: Fasting/ Weight Reduced.

## Supplementary Material

Additional file 1 Table 3 Click here for file
