# Supplementary material for: Regulation of mouse hepatic genes in response to diet induced obesity, insulin resistance and fasting induced weight reduction
Source: Nutr Metab (Lond). 2005 Jun 28;2:15. doi: 10.1186/1743-7075-2-15 (PMC1201568; doi:10.1186/1743-7075-2-15)
Supplement: Additional file 1 — Table 3 [file 1743-7075-2-15-S1.pdf]

Group A

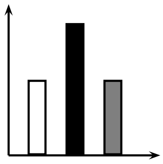

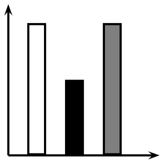

White Bars = Control Mice
Black Bars = High-Fat Mice
Gray Bars = F/ WR Mice

Genes up- or down-regulated by the high-fat diet and normalized to control levels by fasting/ weight reduction.

Gene Name	GenBank #	Control Mice	High-Fat Mice	F/ WR Mice	Biological Role
Crym	NM_ 016669	100%	69%	120%	μ-Crystallin
Cyp2c37	NM_ 010001	100%	30%	95%	Cytochrome P450
Eva	BC015076	100%	162%	119%	Epithelial V-like Antigen
Kcnk8	NM_ 010609	100%	38%	77%	Potassium Channel
Ndph	NM_ 010883	100%	164%	81%	Neuron Function
Pmm1	AK004631	100%	46%	87%	Protein Secretion
Serpina5	NM_ 008785	100%	171%	107%	Serine Protease Inhibitor
Sh3kbp1	AK004636	100%	241%	107%	Signaling
1110034G24Rik	AK004090	100%	45%	80%	Unknown
1700019L13Rik	AK006130	100%	55%	114%	Unknown
4930442L21Rik	NM_ 026253	100%	220%	116%	Unknown
4930579D07Rik	AK016314	100%	42%	72%	Unknown
4932422M17Rik	AK016534	100%	72%	140%	Unknown

continued on next page

Group B

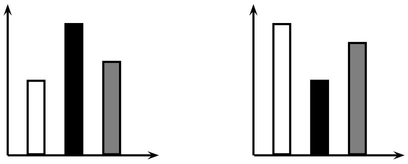

White Bars = Control Mice
Black Bars = High-Fat Mice
Gray Bars = F/ WR Mice

Genes up- or down-regulated by the high-fat diet and partially normalized to control levels by fasting/ weight reduction.

Gene Name	GenBank #	Control Mice	High-Fat Mice	F/ WR Mice	Biological Role
Eif4a2	NM_ 013506	100%	37%	58%	Translation
Fshβ-like EST	AK017593	100%	40%	80%	Hormone
Mup4	NM_ 008648	100%	38%	64%	Secreted Protein
PTP4a2	NM_ 008974	100%	71%	89%	Signaling
RGS3	AF350047	100%	35%	54%	Signaling
Tcam1	NM_ 029467	100%	49%	73%	Adhesion Molecule

Group C

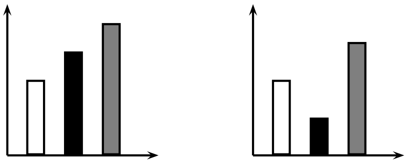

White Bars = Control Mice
Black Bars = High-Fat Mice
Gray Bars = F/ WR Mice

Genes up-regulated by high-fat diet and fasting/ weight reduction, or genes down-regulated by high-fat diet and up-regulated by fasting/ weight reduction.

Gene Name	GenBank #	Control Mice	High-Fat Mice	F/ WR Mice	Biological Role
1500004A08Rik	AK005141	100%	149%	239%	Unknown
2300009A05Rik	AK009046	100%	79%	159%	Unknown
2810055C24Rik	AK012951	100%	64%	127%	Protein Degradation

continued on next page

Group D

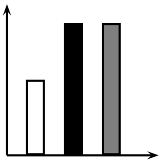

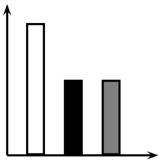

White Bars = Control Mice
Black Bars = High-Fat Mice
Gray Bars = F/ WR Mice

Genes up- or down-regulated by the high-fat diet and fasting/ weight reduction.

Gene Name	GenBank #	Control Mice	High-Fat Mice	F/ WR Mice	Biological Role
Bmp2	NM_ 007553	100%	153%	210%	Development
Copz2	NM_ 019877	100%	266%	521%	Vesicle Trafficking
Fosb	NM_ 008036	100%	231%	297%	Signalling
Gabrr1	NM_ 008075	100%	245%	361%	Receptor
Has3	NM_ 008217	100%	37%	49%	Hyaluronan Synthesis
IL6st	NM_ 010560	100%	154%	144%	Signalling
Rab3c	NM_ 023852	100%	160%	260%	Exocytosis
Ttr	NM_ 013697	100%	63%	44%	Hormone Transport
1110007C24Rik	AK014449	100%	255%	459%	Unknown
2700062B08Rik	NM_ 029838	100%	266%	405%	Putative Collagen-like (CLAC)
3110004A18Rik	AK013988	100%	258%	463%	Unknown
4833414G15Rik	AK019515	100%	162%	257%	Putative Phosphatase
4933432M07Rik	AK017027	100%	223%	191%	Protein Degradation
5730458M16Rik	AK017674	100%	200%	235%	Unknown

continued on next page

Group E

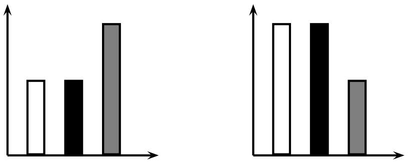

White Bars = Control Mice
Black Bars = High-Fat Mice
Gray Bars = F/ WR Mice

Genes up- or down-regulated by fasting/ weight reduction.

Gene Name	GenBank #	Control Mice	High-Fat Mice	F/ WR Mice	Biological Role
Ctrl	NM_ 023182	100%	121%	327%	Protein Metabolism
Resp18	NM_ 009049	100%	88%	54%	Hormone Secretion
Snrpg	NM_ 026506	100%	89%	199%	Translation
2310034L21Rik	NM_ 025631	100%	123%	225%	Unknown

Group F

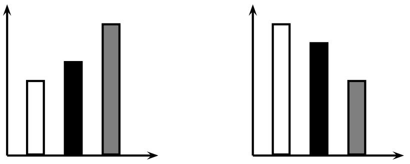

White Bars = Control Mice
Black Bars = High-Fat Mice
Gray Bars = F/ WR Mice

Genes up- or down-regulated by high-fat diet and fasting/ weight reduction.

Gene Name	GenBank #	Control Mice	High-Fat Mice	F/ WR Mice	Biological Role
1700095D18Rik	AK007076	100%	158%	293%	RNA-binding Region
